# Judgment of musical emotions after cochlear implantation in adults with progressive deafness

**DOI:** 10.3389/fpsyg.2015.00181

**Published:** 2015-03-12

**Authors:** Emmanuèle Ambert-Dahan, Anne-Lise Giraud, Olivier Sterkers, Séverine Samson

**Affiliations:** ^1^Unité Otologie, Implants auditifs et Chirurgie de la base du crâne, Assistance Publique Hôpitaux de Paris – Groupe Hospitalier Pitié-SalpêtrièreParis, France; ^2^Laboratoire PSITEC (EA 4072), Neuropsychologie: Audition, Cognition et Action, Department of Psychology, Université de Lille 3Villeneuve d’Ascq, France; ^3^Neuroscience Department, Campus Biotech, University of GenevaGeneva, Switzerland; ^4^Unité d’épilepsie, Assistance Publique Hôpitaux de Paris – Groupe Hospitalier Pitié-SalpêtrièreParis, France

**Keywords:** acquired deafness, cochlear implant, music, emotion, arousal, valence

## Abstract

While cochlear implantation is rather successful in restoring speech comprehension in quiet environments (Nimmons et al., 2008), other auditory tasks, such as music perception, can remain challenging for implant users. Here, we tested how patients who had received a cochlear implant (CI) after post-lingual progressive deafness perceive emotions in music. Thirteen adult CI recipients with good verbal comprehension (dissyllabic words ≥70%) and 13 normal hearing participants matched for age, gender, and education listened to 40 short musical excerpts that selectively expressed fear, happiness, sadness, and peacefulness ( Vieillard et al., 2008). The participants were asked to rate (on a 0–100 scale) how much the musical stimuli expressed these four cardinal emotions, and to judge their emotional valence (unpleasant–pleasant) and arousal (relaxing–stimulating). Although CI users performed above chance level, their emotional judgments (mean correctness scores) were generally impaired for happy, scary, and sad, but not for peaceful excerpts. CI users also demonstrated deficits in perceiving arousal of musical excerpts, whereas rating of valence remained unaffected. The current findings indicate that judgments of emotional categories and dimensions of musical excerpts are not uniformly impaired after cochlear implantation. These results are discussed in relation to the relatively spared abilities of CI users in perceiving temporal (rhythm and metric) as compared to spectral (pitch and timbre) musical dimensions, which might benefit the processing of musical emotions (Cooper et al., 2008).

## INTRODUCTION

Most hearing people consider music to be a source of pleasure, which certainly constitutes the main motivation for listening and playing music. In post-lingual progressive deafness, however, not only is oral communication compromised, but also musical pleasure and the social enjoyment of music (Gfeller et al., 2002b,c). When hearing loss becomes too profound to be alleviated by conventional hearing aids, cochlear implantation is often proposed to restore hearing. Cochlear implants (CIs) consist of an array of electrodes that are inserted inside the cochlea, and which directly stimulate the auditory nerve fibers by converting the acoustic information from environmental sounds into electrical pulses. The temporally and spectrally coded information contained in the CI signal is then transmitted to the auditory cortex.

While CIs are rather successful with respect to restoring speech comprehension in quiet environments (Nimmons et al., 2008), music perception and enjoyment remains challenging for cochlear implantees (Mirza et al., 2003; Migirov et al., 2009; Brockmeier et al., 2011). CI users generally tend to minimize their exposure to music (Lassaletta et al., 2008), either because their relation to music has been altered during the period of profound deafness, or following implantation, whereby the electric nature of the signal delivered by CI dramatically changes musical sensations (Smith et al., 2002; Kong and Zeng, 2006; Gfeller et al., 2007; Filipo et al., 2008; Nardo et al., 2008). Most CI recipients exhibit poor performance in musical processing (Leal et al., 2003; Looi et al., 2008). One notable exception in the literature depicts the case of a 30-year-old non-professional musician who displayed excellent musical perception abilities after bilateral cochlear implantation following 3 years of deafness that was caused by inner ear autoimmune disease (Maarefvand et al., 2013). Surprisingly, this person presented no deficit in pitch direction discrimination with a threshold to half of a semitone, and no impairment in melody and timbre recognition, which was tested using the Clinical Assessment of Music Perception tests (CAMP, Kang et al., 2009). Most post-lingually deaf adult CI users, however, display severe difficulties in pitch discrimination and in musical timbre or familiar melody recognition (Gfeller et al., 2002a; Kong et al., 2004; McDermott, 2004; Looi et al., 2008; Kang et al., 2009) relative to normal hearing (NH) persons. When exposed to the Montreal Battery for Evaluation of Amusia (Peretz et al., 2003), CI users have been found to perform at near chance level on pitch–based tests (Scale, Contour, and Interval), yet obtained relatively higher scores on temporal-based tests (Rhythm and Meter), which suggests that they have no deficit in rhythm-based perception (Gfeller et al., 2000; Looi et al., 2008) or tempo discrimination (Kong et al., 2004). Temporal processing capacity is determined by the sampling rate of the implants, which is generally very high and thus yields minimal impairment. For pitch perception, however, the limitations are both more drastic and more complex, as they result from the combination of constraints on the place- and rate-coding of pitch. One fundamental limitation of pitch place-coding in CI users is that the spread of excitation produced by electrical stimulation is less focused than in the normal ear (Macherey and Carlyon, 2014). There is an upper rate pitch limit of around 300 Hz on most (Shannon, 1983; Zeng, 2002) but not all subjects (Kong et al., 2009; Penninger et al., 2014). Several attempts have been made so far to improve this temporal pitch limit. The two most promising attempts to overcome this limitation are the use of asymmetric pulses (Macherey et al., 2011), and stimulating on multiple electrodes (Venter and Hanekom, 2014; Penninger et al., 2015).

Paradoxically, deaf persons specify music enjoyment as a major motivation for getting an implant, beyond the expected benefit on speech perception and communication (Gfeller et al., 2002b). One third of CI candidates resort to cochlear implantation for the mere purpose of being able to listen again to music (Kohlberg et al., 2013) and many of them consider that improving music perception abilities will enhance their quality of life (Drennan and Rubinstein, 2008). Results from two surveys (Lassaletta et al., 2008; Migirov et al., 2009) completed by 65 and 53 individuals, respectively, indicate that post-lingually deaf CI users continue to listen to music, albeit at a generally diminished exposure duration. Although classical Western music heavily relies on accurate pitch perception, listening to music remains a pleasant experience for some CI listeners (Kohlberg et al., 2013). This finding suggests that CI users might recognize emotions conveyed by music despite their impaired pitch perception.

However, there are still very few objective data on the capacity of CI users to perceive musically evoked emotions. Music appreciation in CI patients has essentially been explored using self-report questionnaires that characterize listening habits and the enjoyment of music before and after cochlear implantation. A single study reports both emotional judgments and music perception abilities in six post-lingual CI adult users and six NH participants (Rosslau et al., 2012). CI users were able to perceive various degrees of arousal in famous movie soundtracks, and did not show the typical difficulties in pitch, rhythm, and harmony perception, as measured by the standard MuSIC perception test battery (Brockmeier et al., 2011). Although these findings suggest that CI users are still able to perceive arousal conveyed by music, one should remain cautious about the interpretation of these results given the small number of participants and the familiarity of the tested musical material.

In order to address the ability for CI users to perceive emotions conveyed by music, we examined how post-lingually deaf CI users evaluate musical excerpts composed with the intention of evoking fear, peacefulness, happiness, and sadness, and compared their results with those of NH subjects. Participants performed three different judgment tasks. The first is an emotional categorization judgment that requires the listener to assess the degree of threat, peacefulness, happiness, and sadness in musical excerpts. The second and third judgments involve the rating of emotional dimensions: arousal and valence. Participants evaluated the quality of music excerpts in terms of arousal (from ‘relaxing’ to ‘stimulating’), and in terms of valence [from ‘pleasant’ (positive) to ‘unpleasant’ (negative)]. Therefore, musical emotion perception was assessed using both a categorical and a dimensional approach. Admittedly, while music can induce basic emotions such as happiness, sadness, anger, or fear, the categorical approach is insufficient to accurately portray the richness of emotional feelings related to musical experience (Bigand et al., 2005). A combined approach accomodates Russell’s (1980) model of affect, which stipulates that emotions can be represented in a two-dimensions emotional space defined by arousal and valence (Eerola and Vuoskoski, 2011).

The objective of this study was to assess the abilities of CI users to perceive and judge emotions in music, as well as the preservation of emotional skills in the case of progressive deafness. Given the music perception disturbances in CI users (e.g., Cooper et al., 2008; Kang et al., 2009), we predicted that these persons would show deficits in recognizing the four cardinal emotions (happiness, peacefulness, fear, and sadness) of the musical excerpts. In particular, we hypothesized that emotional labels with disctinctive rapid tempos such as happiness will be better recognized in musical excerpts due to the specific use of temporal code for pitch processing (Macherey and Carlyon, 2010). In turn, peacefulness, fear, and sadness were hypothesized to be more poorly recognized because of their similar and characteristically slower tempo with melodic lines lying in the medium pitch range. Since it has been shown in NH individuals that arousal judgments rely mainly on spectral cues, which are badly transmitted by CIs, we predicted that CI users would be also impaired in judging emotional arousal in musical excerpts. Finally, considering that CI patients continue to listen to music, we conjectured that they would still experience pleasure and appreciate music, and therefore that they might be less affected in rating emotional valence than they would rating arousal.

## MATERIALS AND METHODS

### PARTICIPANTS

Thirteen French-speaking patients with severe to profound post-lingual progressive sensorineural hearing loss were tested at least 1 year after unilateral cochlear implantation. Most patients had a controlateral hearing aid (HA; *n* = 11) and the remaining participants (*n* = 2) had no controlateral HA. All participants used oral communication rather than sign language and had good speech perception performances (dissyllabic word recognition ≥70%) after cochlear implantation, at the time of testing. None had a history of neurological or psychiatric illness. All participants were tested at the ENT department (Beaujon hospital, Paris). A group of 13 normal-hearing (NH: 9 female/4 male; mean age = 57.0 years, SD = 11.1; mean duration of education = 17.1 years, SD = 3.0) controls matched to the CI patients in terms of sex, age and education (CI: 9 female/4 male; mean age = 57.8, SD = 11.5; mean duration of education = 15.2 years, SD = 1.8) were also tested. None of the participants were musicians except for one CI patient (CI 13). Demographic, clinical, and language data of the CI users are presented in **Table 1**. All participants gave their written informed consent before being tested in accordance with the Declaration of Helsinki.

**Table 1:** Demographic, clinical and language data for each patient of the CI group (CI, cochlear implant; HA, hearing aid; S/N, signal to noise ratio).

Participant	Sex	Age	Education (years)	Cause of deafness	Degree of loss (dB)	Profound deafness duration (years)	Side of CI	CI sound processor	Coding strategy	CI Duration (months)	Dissyllabic words with HA (%) before CI	Dissyllabic words with CI alone (%)	Sentences with CI S/N = 10 dB (%)	Musical experience
Post-CI 1	Female	68	20	Otosclerosis	Left = 112 Right = 89	10–20	L	Freedom (Cochlear)	ACE/900 Hz	12	60	90	53	No
Post-CI 2	Female	74	15	Hereditary	Left = 120 Right = 120	>20	L	Freedom (Cochlear)	SPEAK/250Hz	36	50	80	62	No
Post-CI 3	Male	39	16	Unknown	Left = 120 Right = 102	0–10	R	Freedom (Cochlear)	ACE/900 Hz	12	0	90	93	No
Post-CI 4	Female	76	15	Unknown	Left = 74 Right = 93	>20	R	Freedom (Cochlear)	ACE/900 Hz	24	70	90	88	No
Post-CI 5	Male	59	14	Traumatic	Left = 95 Right = 120	0–10	L	Harmony (Ad.Bion.)	ACE/900 Hz	51	100	100	78	No
Post-CI 6	Female	63	15	Schwanomma	Left = 120 Right = 81	0–10	R	Freedom (Cochlear)	ACE/900 Hz	20	0	70	64	No
Post-CI 7	Female	59	12	Otosclerosis	Left = 88 Right = 53	10–20	R	Freedom (Cochlear)	ACE/900 Hz	18	90	100	40	No
Post-CI 8	Female	55	14	Neuropathy	Left = 80 Right = 107	0–10	R	Freedom (Cochlear)	ACE/900 Hz	24	10	100	97	No
Post-CI 9	Female	52	14	Unknown	Left = 77 Right = 85	0–10	L	Opus 2 (Medel)	HiresS/Fidelity 120	18	50	80	77	No
Post-CI 10	Female	39	15	Unknown	Left = 120 Right = NA	0–10	L	Freedom (Cochlear)	ACE/900 Hz	120	20	90	81	No
Post-CI 11	Female	54	16	Crohn disease	Left = 62 Right = 120	0–10	R	Freedom (Cochlear)	FSP	57	60	80	100	No
Post-CI 12	Male	50	16	Menière	Left = 120 Right = 48	0–10	L	Freedom (Cochlear)	ACE/900 Hz	48	50	90	60	No
Post-CI 13	Male	63	16	Menière	Left = 45 Right = 85	0–10	R	Harmony (Ad.Bion.)	HiresS/Fidelity 120	18	100	60	0	Yes

Mean	–	57.8	15.2	–	–	–	–	–	–	35.2	50.8	83.8	68.7	–
SD	–	11.5	1.8	–	–	–	–	–	–	29.6	35.0	11.9	27.4	–

### PRELIMINARY MOOD ASSESSMENT

In order to ensure that the evaluation of emotions conveyed by music was not biased by transient or chronic mood disturbances, which are often observed in deaf patients, subjects completed two questionnaires. The State and Trait Anxiety Inventory (STAI: Spielberger, 1983) and the Profile of Mood States (POMS: McNair et al., 1992) were administrated before the emotional rating task. The STAI is composed of two scales: the trait STAI to assess the general level of anxiety, which is presumed to be stable over time, and the state STAI to assess the present level of anxiety. The POMS is composed of 30 emotional adjectives and the participants had to rate how they describe their current mood (from 0 *not at all* to 4 *extremely*). Six emotional scales can be derived from these responses: Tension, Depression, Anger, Vigor, Fatigue, and Confusion. As report in **Table 2**, there was no difference between the two groups of participants in either of these scores.

**Table 2:** Results for mood questionnaire for CI and normal hearing (NH) participants.

	Groups		Mann–Whitney
	NH	CI		
**STAI**
Trait anxiety	31.69 ± 1.58	36 ± 1.79	*U* = 71.5, *p* > 0.05
State anxiety	37.38 ± 1.67	34 ± 2,60	*U* = 81.5, *p* > 0.05
**POMS**
Anger	3.23 ± 0.85	3.54 ± 0.98	*U* = 81.5, *p* > 0.05
Anxiety	3.62 ± 0.58	3 ± 0.74	*U* = 65, *p* > 0.05
Depression	3.08 ± 0.56	2.62 ± 0.84	*U* = 65.5, *p* > 0.05
Confusion	5.15 ± 0.62	4.77 ± 0.82	*U* = 68.5, *p* > 0.05
Vigor	12.77 ± 0.61	13.77 ± 1.28	*U* = 61, *p* > 0.05
Fatigue	3.31 ± 0.52	3.85 ± 1.01	*U* = 83.5, *p* > 0.05

### STIMULI

The musical material consisted of 40 excerpts that conveyed four intended emotions (happiness, *n* = 10; sadness, *n* = 10; threat, *n* = 10, and peacefulness, *n* = 10). The intended emotion, valence, and arousal of each selected musical excerpt have been validated by previous studies (Gosselin et al., 2005; Vieillard et al., 2008). Musical excerpts were composed following the rules of the Western tonal system, and were based on a melody with an accompaniment. The stimuli had a regular temporal structure with the exception of a few fearful excerpts. The happy excerpts were written in a major mode at an average rapid tempo, the melodic line lying in the medium–high pitch range, and the pedal was not used. In contrast, the sad excerpts were written in a minor mode at an average slow tempo, and the pedal was used. The peaceful music was composed in a major mode, had an intermediate tempo, and was played with pedal and arpeggio accompaniment. Most fearful excerpts were regular and consonant with various tempos that ranged from slow to rapid, and were composed of minor chords on the third and sixth degrees, which implies the use of accidentals. Only few excerpts had irregular rhythms and were dissonant. Among the 10 fearful musical excerpts, ratings of overall dissonance (from 1 for “not dissonant at all” to 5 for “very dissonant”) indicated that all the stimuli were not considered as dissonant, with scores ranging from 1 to 3. For the ratings of rhythmic regularity of these fearful excepts (from 1 for “fairly regular” to 5 for “irregular”), six stimuli were judged to be irregular, with scores ranging from 3 to 5, while four of them were judged to be more regular, with scores from 1 to 2. All excerpts were computer-generated and recorded in a piano timbre with a mean duration of 12 s. Examples can be heard at .

### PROCEDURE

The musical excerpts were presented in a pseudo-randomized order using Presentation software (Neurobehavioural Systems Inc., San Pablo, CA, USA) and at a comfortable listening level of 70 dB SPL. The stimuli were delivered via two loudspeakers (Logitech X-140, RMS total power of 5 W, bandwidth 80 Hz–18 kHz) that were positioned on each side of the video monitor, which allowed us to test CI subjects under optimal listening conditions, i.e., binaural hearing if they had a contralateral HA, or with their CI only.

After the presentation of each musical excerpt, participants performed three different judgments. In the first, they rated to what extent the music expressed threat, peacefulness, happiness, and sadness on four rating scales, whereby 0 corresponded to ‘absent’ and 100 to ‘present.’ The second and third judgments required participants to rate the arousal (from ‘relaxing’ to ‘stimulating’) and valence (from ‘unpleasant’ or ‘negative’ to ‘pleasant’ or ‘positive’) of each musical excerpt.

Participants were explicitly asked not to judge their own felt emotion but to rate the emotion conveyed by the musical excerpt. The stimuli were presented only once and no feedback was given. Ratings along the visual analog scale were displayed on a computer screen with identical unmarked horizontal lines and numerical labels at each extremity (0–100).

Prior to the experiment, a training session was carried out in order to ensure that each participant recognized the targeted emotional category (fear, sadness, happiness, and peacefulness) by associating short sentences to the most appropriate emotional label (e.g., for happiness: ‘Eric had just won the lottery’). The comprehension of the terms ‘valence’ and ‘arousal’, was also verified by rating four situations (e.g., ‘Sophie has just received a wonderful travel for her wedding’ was supposed to be highly positive and arousing). Feedback was presented to ensure the appropriate use of the two scales.

## RESULTS

### CATEGORICAL JUDGMENTS

Since participants were allowed to indicate a combination of perceived emotions (happiness, fear, sadness, and peacefulness) by providing a graded judgment for each, we first derived, for each participant, the dominant emotion attributed to each musical excerpt by selecting the rating scale that had received the maximal rating. When the highest rating was given for more than one emotion, e.g., when a participant judged a musical excerpt to express both surprise and fear to the same degree, it was considered ambivalent and was not included in the analysis.

To compare emotional labels assigned by CI users and by NH participants, we calculated correctness scores for each participant using a proportional approach as proposed by Heberlein et al. (2004): each response was given a correctness score based on the proportion of subjects in the NH group that gave that response (minimum of the correctness score = 0; maximum = 1). Higher correctness scores corresponded to answers that were chosen a large number of times by NH participants, whereas lower correctness scores corresponded to answers that were less frequently chosen by NH participants. However, NH participants used both ears to make their judgments.

A correctness score of ‘zero’ corresponded to responses that had never been given by NH participants. Correctness scores are useful because any given emotional musical excerpt can be perceived as expressing more than one single emotion, inducing variability in the labeling by normal listeners. In other words, such a proportional method takes into account the type of errors, considering that some errors are more acceptable than others. For example, a peaceful musical excerpt can also express some sadness, and it is less inaccurate to judge this excerpt as expressing sadness than happiness even if sadness is not the intended emotion. Considering such a graduation among the type of errors, using correctness scores permitted a refinement of the analysis.

**FIGURE 1:**
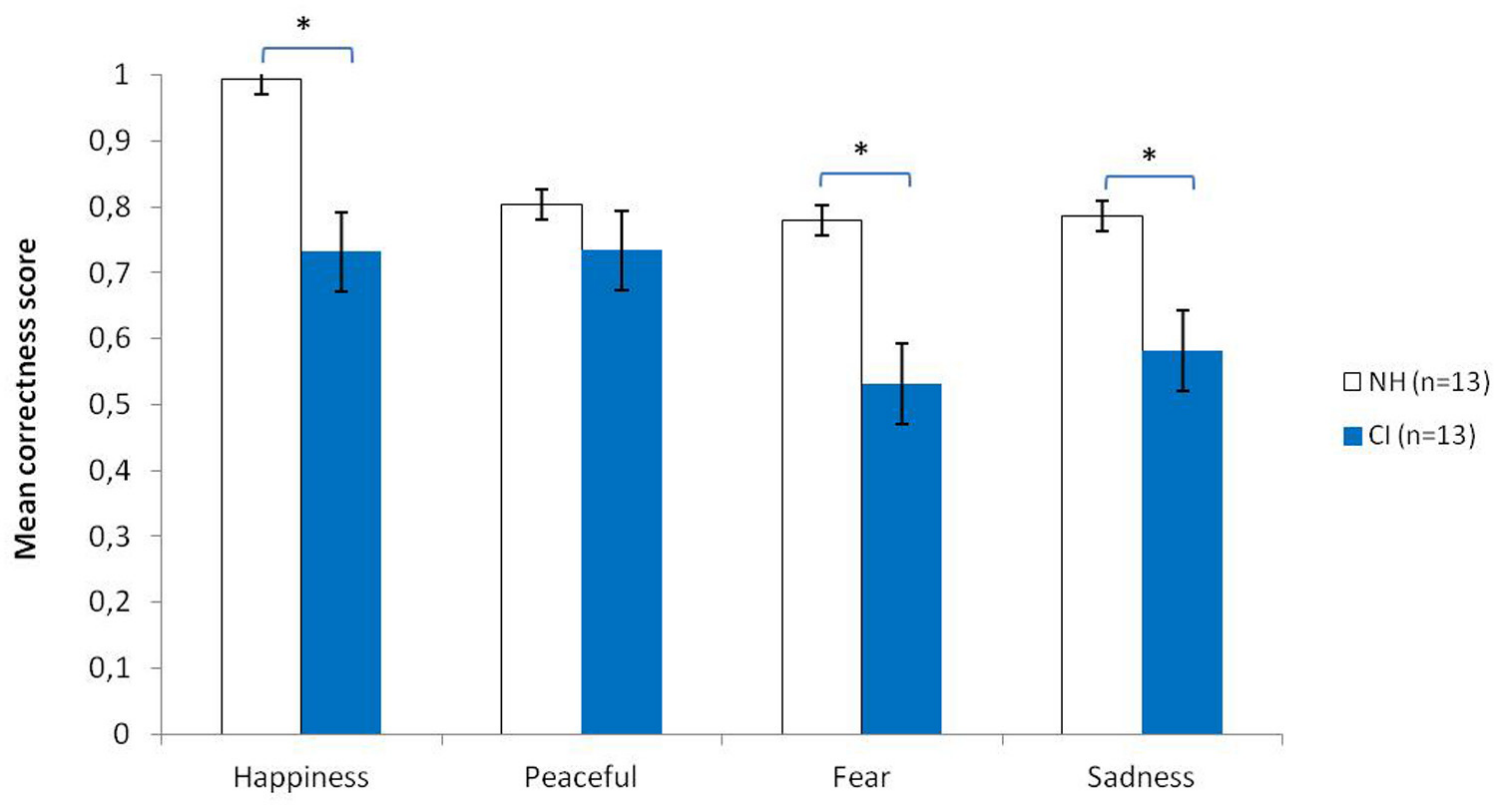
**Mean correct scores of the two groups of participants (NH, normal hearing controls; CI, cochlear implanted users) as a function of the four intended emotions (bars represent the SE of the mean).** Asterisk means significant difference.

As can be seen in **Figure 1**, CI patients’ judgments differed from those of NH participants, especially for happiness, fear, and sadness. Happy, scary, and sad music were indeed less well recognized by CI patients (happy = 0.73; scary = 0.53; sad = 0.58) than by NH participants (happy = 0.99; scary = 0.78; sad = 0.78). Since the criteria for variance homogeneity were not met, we used non-parametric analyses to compare average correctness scores in CI and NH groups as a function of the intended emotion. Mann–Whitney *U*-tests revealed that CI participants were less accurate in recognizing happiness than were NH participants (*z* = -2.54; *P* < 0.05), fear (*z* = -3.36; *P* < 0.001) and sadness (*z* = -2.59; *P* < 0.01), but there was no difference for peacefulness (*p* > 0.05). However, all CI participants performed well above chance level (25%).

### JUDGMENTS OF VALENCE AND AROUSAL

**FIGURE 2:**
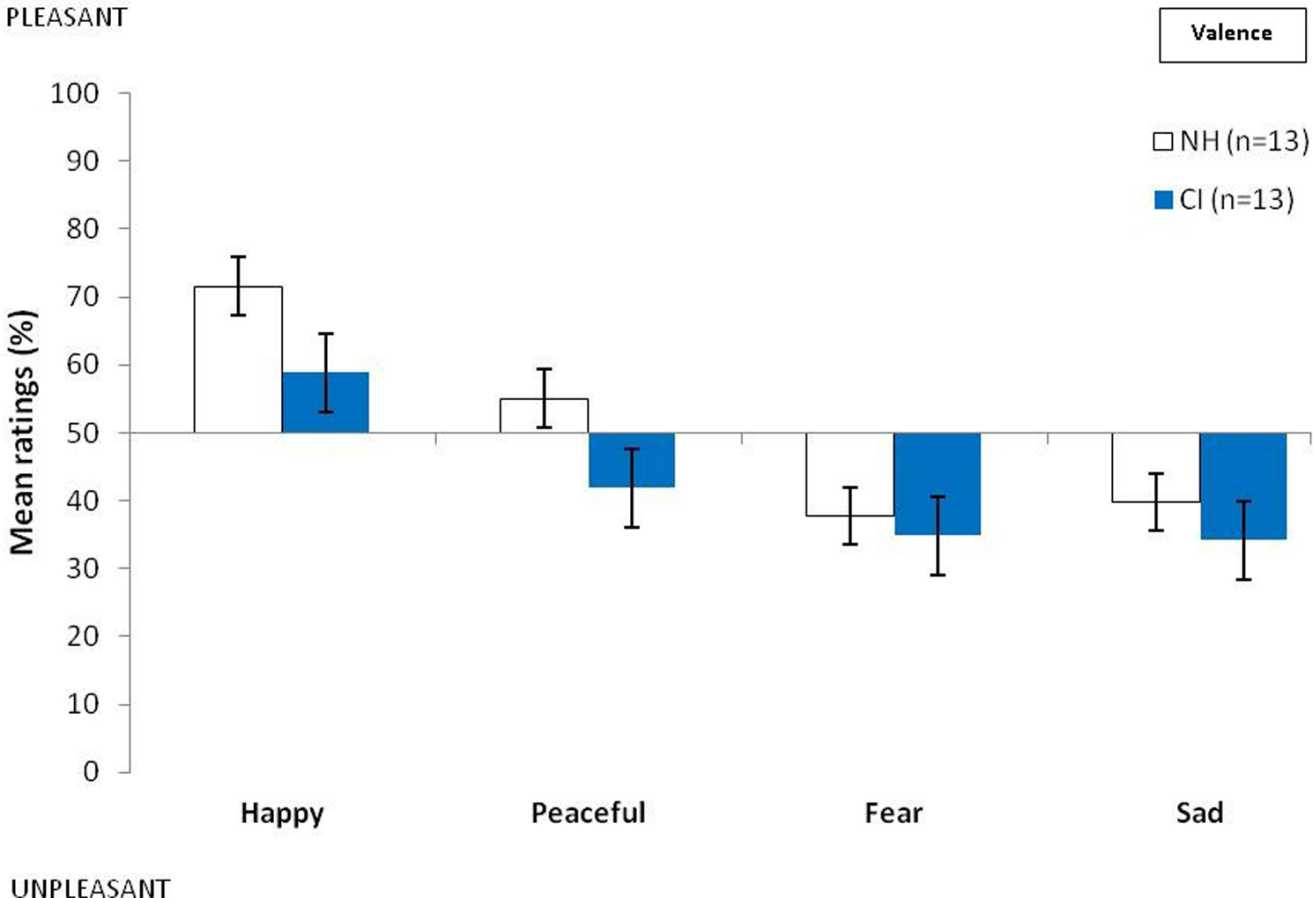
**Mean ratings of the two groups of participants in judging valence of musical excerpts as a function of the intended emotions and groups (NH, normal hearing controls; CI, cochlear implanted users; bars represent the SE of the mean)**.

**FIGURE 3:**
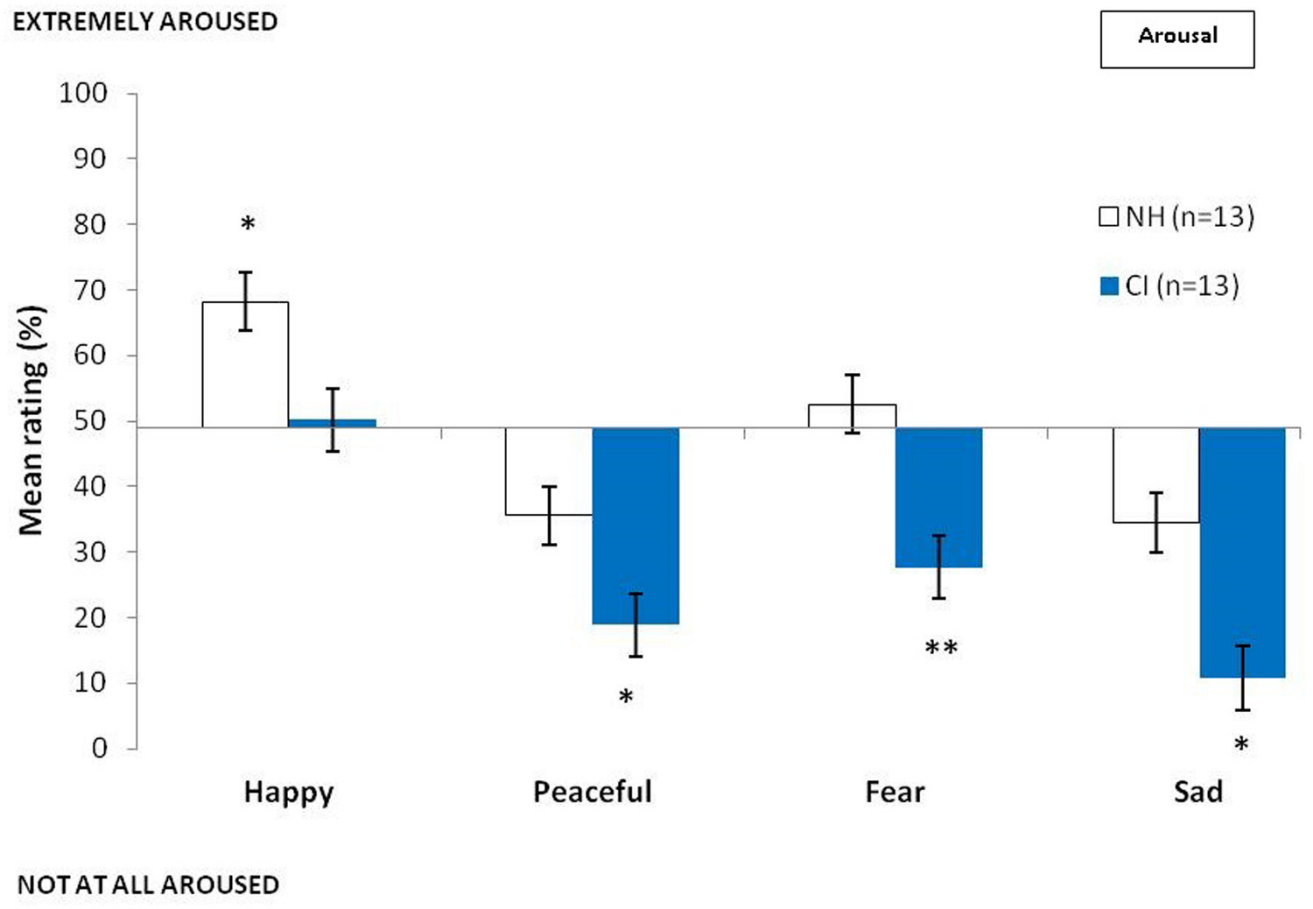
**Mean ratings of the two groups of participants in judging arousal of musical excerpts as a function of the intended emotions and groups (NH, normal hearing controls; CI, cochlear implanted users; bars represent the SE of the mean)**.

The ratings of valence and arousal obtained for each emotional category by the CI and NH groups are presented respectively in **Figures 2 and 3**. Mann–Whitney *U*-tests revealed there to be no group difference between the valence scores for the different emotions (happy: *z* = -1.31; *P* > 0.05; peaceful: *z* = -1.72; *P* > 0.05; fearful: *z* = -0.44; *P* > 0.05; sad: *z* = -0.90; *P* > 0.05). However, we found group differences between the ratings of arousal for happy (*z* = -2.07; *P* < 0.05), peaceful (*z* = -2.28; *P* < 0.05), fearful (*z* = -3.31; *P* < 0.001), and sad musical excerpts (*z* = -3.00; *P* < 0.01), whereby CI users judged musical excerpts to be less arousing than did NH participants.

### INDIVIDUAL ANALYSES

To further explore the results for musical emotions, we compared individual correctness scores between CI and NH participants (Crawford and Garthwaite, 2007), using the mean score of the four intended musical emotions. The results showed that seven CI participants (CI 1, 6, 7, 8, 9, 11, and 12) out of 13 were significantly impaired in categorical judgments of musical emotions (all *p*s < 0.05). These impaired patients were on average 57 years old [50–68] and the mean duration of CI use was 28 months [12–57]. By contrast, the six CI participants that did not show any deficit (CI 2, 3, 4, 5, 10, and 13), who had an average age of 58 years [39–76], had a mean duration of CI use of 44 months [12–120].

### EMOTIONAL JUDGMENTS OF MUSIC AND LANGUAGE PERFORMANCES

Spearman’s correlation between language performance in noise with CI (global score of sentence recognition with a signal to noise ratio of 10 dB) and emotional judgments of musical excerpts was computed for CI users. Language performance in noise was correlated with the global mean correctness score of the four intended musical emotions (ρ = 0.60, *p* < 0.05) but not with the perception of arousal (ρ = -0.29, *p* > 0.05).

## DISCUSSION

The aim of our study was to test how CI users with acquired post-lingual progressive deafness perceive emotions conveyed by music. We used an emotional rating task in which participants evaluated the amount of the four cardinal emotions (peacefulness, happiness, sadness, and fear) present in musical excerpts on visual analog scales, using the same stimuli as Gosselin et al. (2005) and Vieillard et al. (2008). We also assessed the perception of arousal and valence of the same musical excerpts in order to specify the ability CI users to experience emotional dimensions.

Our results show that CI participants performed less accurately than NH participants in recognizing happy, sad, and scary music, with scores that were, however, well above chance level. By contrast, they performed just as well as NH controls on recognizing peaceful musical excerpts. Altogether, these results suggest that emotional judgments of musical excerpts were not uniformly affected by artificial hearing with a CI. We also found there to be a deficit in perceiving emotional arousal, CI users being less excited by music than NH listeners. Conversely, rating of emotional valence was not impaired in CI users. Given that none of the participants were able to perform such judgments before cochlear implantation due to profound deafness, these results demonstrate the benefit of CIs for perceiving musical emotions and for discriminating between pleasantness and unpleasantness in music. These findings give experimental support to the observation that CI users can still be attracted to music and can enjoy music listening (Kohlberg et al., 2013), even if they cannot access the whole spectrum of musical emotions.

The impairments we observed in musical emotion ratings were not accounted for by depressive disorders that often affect profoundly deaf subjects (Hallam et al., 2006; Garnefski and Kraaij, 2012). The STAI (Spielberger, 1983) and POMS (McNair et al., 1992) questionnaires revealed there to be no difference between CI and NH participants, which indicates that depression or anxiety disturbances did not interfere with judgments of musical emotion. Moreover, as all patients were able to understand the emotional labels used in the tasks, the deficits in recognizing happy, sad, and scary music seen here cannot be explained by difficulties in language comprehension or by a general emotional deficit, as recognition of peaceful music and perception of valence remained intact.

The deficits in emotional judgments shown here can presumably be attributed to the poor transmission of pitch cues and particularly fundamental frequency (F0) by the device. Most current CIs less efficiently transmit the fine structural key aspects of musical sounds than does a physiological cochlea, and convey coarse spectral cues. One reason for this is that a CI device has a limited number of wide filter bands with fixed center frequencies that may hinder complete resolution of the lower harmonics of complex musical sounds. However, pitch may also be perceived through sound rate (periodicity pitch), although this mechanism is limited to rates up to 300–400 Hz. The deficit in place coding cues, with reduced excitation in the most apical part of the cochlea, the mismatch between the rate of stimulation and the cochlear tonotopy (Looi et al., 2012), and the insufficiency of fine temporal variations processing, all concur to limit pitch perception in CI users (Carlyon et al., 2014) and prevent a full harmonic processing. Limitations of CI transmission that produce impairments in pitch, timbre, and melody perception (i.e., Gfeller et al., 2002a, 2008; McDermott, 2004) can specifically affect the recognition of happy, sad, and scary music by CI users. Yet, as pitch discrimination remains fairly good when stimuli are far apart in the spectral domain (Gfeller et al., 2002a; Kong et al., 2004; Looi et al., 2004; McDermott, 2004), CI users may retain the ability to distinguish emotions in music, such as telling apart happiness from peacefulness or sadness, whose melodic lines are in the medium–high pitch and medium–low pitch ranges, respectively.

The preserved ability to recognize some musical emotions can also be explained by the use of timing and rhythmic cues that CI users remain able to process. Perception of rhythm in music is related to the perception of the duration of sounds and intervals between sounds that CI users perceive (McDermott, 2004). Rhythmic differences are hence encoded as temporal gaps or amplitude modulations, or both (Shannon, 1989, 1992), and CI listeners have temporal processing abilities with performances for periodic pulse trains’ rate discrimination tasks based on perceived pitch up to 300 pps (Moore and Carlyon, 2005). Therefore, CI recipients perform as well as NH adults in rhythm discrimination tasks (Gfeller et al., 2000; Kong et al., 2004; Looi et al., 2008) and tempo perception (Kong et al., 2004).

Given that a rapid tempo is often associated with a positive but also arousing emotion such as happiness, while a slower tempo often conveys a more negative and less arousing emotion such as sadness, temporal cues could also play a role in perceiving emotional valence and arousal. However, it remains difficult to explain why CI listeners can rate emotional valence while at the same time being less sensitive to arousing stimuli than NH participants. Gingras et al. (2013) proposed that emotions in music are conveyed by two types of underlying cues: universal acoustic cues (i.e., intensity, pitch, and tempo) and culturally determined cues that are associated with a specific musical system such as the Western common-practice tonality. Before their hearing loss, CI users have experienced music listening in their everyday life, and have thus been exposed to the use of both types of cues. Due to the preceding period of deafness, CI listeners might continue to rely on cultural cues to compensate for difficulties in processing acoustic cues, being therefore able to perceive emotional valence in music. It is also possible that this ability could be partly explained by their strong motivation to feel pleasantness in music. Preservation of valence judgment could be explained by the search of emotional reward in music, and by the strong motivation of CI users to retrieve normal everyday emotional life, including the social sharing of emotions. Considering that CI users continue to listen to music, we may conjecture that they are still experiencing musical pleasure and appreciation, and that they might be able to feel musical pleasantness through emotional valence, even though the exact cues they use remain to be clarified.

In agreement with our predictions, but in contradiction with Rosslau et al.’s (2012), we found that CI listeners were impaired in arousal judgments. The apparent discrepancy between the studies might be explained by methodological differences. Although the authors used a simple task in which subjects rated three levels of arousal in familiar musical excerpts that all had positive valence, the lack of significant results could be attributed to the small number of participants included in their study (six CI users). To interpret the arousal perception deficit, some authors have invoked the crucial role of intensity cues as musical emotions vectors, positive association between sound intensity and arousal induction in subjective ratings being well documented (Scherer, 1989; Ilie and Thompson, 2006). However, recent evidence suggests that spectral features are more important than intensity cues in arousal rating (Gingras et al., 2013), as the manipulation of these acoustic cues does not have the same effect on music-induced arousal and pleasantness feelings. To validate their hypothesis, non-musician participants were asked to rate subjective arousal and valence (pleasantness) of original vs. amplitude-normalized loudness-matched musical excerpts. Although the manipulation of intensity did not affect the subjective rating of arousal and valence, spectral features (spectral flux and entropy) modulated the perception of arousal in both original and amplitude-normalized loudness matched musical excerpts. This suggests that arousal judgment depends on spectral properties rather than on intensity features. In addition, the authors found that the proportion of variance explained by basic acoustic features is higher for arousal than for valence ratings of musical excerpts. Based on these findings, they concluded that arousal induction could correspond to a bottom–up process related to the physical characteristics of the stimulus (spectral information) whereas valence induction may be more culturally determined in the context of musical emotions. We may therefore propose that difficulties in processing spectral properties of sounds observed after cochlear implantation in post-lingually deaf persons should have a more deleterious impact on arousal than on valence ratings. In addition, according to Brunswick’s (1957) concept assuming that a listener can infer arousal levels from the available inter-correlated cues (rhythmic, tonal, and spectral features), when a more efficient cue is not available, the poor transmission of spectro-temporal redundancy cues by the CI could also be responsible for the impairments of CI users in rating arousal of musical excerpts.

Impairments in arousal judgments in CI subjects may also be considered by opposing the emotivist approach, which focuses focusing on the emotional feelings to the cognitivist approach centered on the perception of emotions in which recognition can operate without association with a subjective feeling (Krumhansl, 1997). This distinction may explain, at least in part, the relative preservation of valence judgment abilities along the cognitive’s view, which presupposes that the pleasant or unpleasant character of a piece of music could be perceived without being felt. Conversely, arousal judgment could be based on the subjective experience of emotional intensity, which could be limited due to the progressive limitation of social interactions in case of progressive deafness. Another possibility is that our results may have been contaminated by the existence of a methodological bias, since we did not counterbalance the order of ratings performed after the presentation of each musical excerpts (categorical judgment was always followed by arousal and valence ratings). However, it remains difficult to understand why this order would have only benefited the last emotional rating of each musical excerpt considering that we found no correlation between the three types of ratings. Moreover, each of them involved very different emotional judgments.

Finally, we found that emotional recognition of musical excerpts, but not perception of arousal, was correlated with sentence recognition with a signal to noise ratio of 10 dB. This finding suggests that recognition of emotional categories and the rating of arousal do not depend on the same variables. Similarly to the perception of emotional features in music, speech perception in noise requires the ability to use pitch cues to separate information from the background, as shown by Qin and Oxenham (2003). Poor pitch perception abilities in CI patients with small benefit for speech perception in noise could partly explain their impairment in recognizing emotional categories of musical excerpts. This impairment could in turn be explained by the lack of pitch coding in CI speech processing strategies conveying more efficiently envelope information than fine (spectral and temporal) structure information (Cullington and Zeng, 2011). Inspection of individual data also showed CI users that present difficulties in recognizing emotion also had shorter post-CI duration and less practice with the device, (except for one CI user who was a former musician) than non-impaired CI users. While these results confirm the impact of the electric nature of the CI signal on musical emotional judgments, they also show that there is some plasticity in the recovery of these auditory functions that are involved in the recognition of musical emotions. In summary, emotional judgment in music could be seen as a complex task that requires good pitch perception, as for speech-in-noise perception with concurrent talkers, and time to adapt to the device.

## CONCLUSION

This study examined for the first time emotional judgments for music in CI adult recipients with progressive sensorineural hearing loss. We tested emotion recognition and the perception of arousal and valence in music, and showed that CI listeners were impaired in judging emotions conveyed by music, even though their performance remained above chance. This finding suggests that cochlear implantation does partially allow for musical emotion processing, and that despite their sensory impairment, CI users can appreciate listening to music, and perceive its valence. The relatively spared abilities of CI listeners to judge emotional valence, as compared to arousal, and to recognize peacefulness in musical excerpts confirm previous observations indicating that CI users can use temporal acoustic cues to process music (Kong et al., 2004). We therefore propose that the larger improvement in processing temporal (rhythm and metric) than spectral (pitch and timbre) cues that follows cochlear implantation (Cooper et al., 2008) contributes to the regain the processing of musical emotions. Taken together, these results demonstrate the benefit of cochlear implantation for emotional perception in music. Although CI users remain impaired relative to NH persons in judging emotional categories and in perceiving arousal in music, they may retrieve the ability to enjoy music listening through the ability to judge emotional valence.

## Conflict of Interest Statement

The authors declare that the research was conducted in the absence of any commercial or financial relationships that could be construed as a potential conflict of interest.
